# Upwelling and eddies affect connectivity among local populations of the goldeye rockfish, *Sebastes thompsoni* (Pisces, Scorpaenoidei)

**DOI:** 10.1002/ece3.3993

**Published:** 2018-04-02

**Authors:** Hyo Jae Yu, Jin‐Koo Kim

**Affiliations:** ^1^ Department of Marine Biology Pukyong National University Busan Korea

**Keywords:** dispersal barrier, interspecific hybridization, microsatellite loci, mitochondrial DNA, population structure

## Abstract

The goldeye rockfish, *Sebastes thompsoni*, commercial rockfish catch in the Northwest Pacific Ocean, may influence its population structure. To clarify the population genetic structure of Korean *S. thompsoni* and its degree of hybridization with the most close species, *Sebastes joyneri*, we analyzed a mitochondrial (mt) DNA control region and eleven polymorphic microsatellite (ms) loci. *S. joyneri* individuals were clearly distinguished from *S. thompsoni* by the mtDNA control region and ms loci results, with single interspecific hybridization between two species suggesting no impact on genetic structure of *S. thompsoni*. Analysis of mtDNA revealed no population structure within *S. thompsoni*, suggesting the survival of a single population in southern refugia during the glacial period. The ms loci results, in contrast, showed two genetically distinct clusters within *S. thompsoni*: One was predominant throughout Korean coasts (from the Yellow Sea, via the Korea Strait to the East Sea); the other was predominant at Dokdo Island in the East Sea; and both occurred in similar ratios at Wangdolcho Reef in the East Sea. A possible factor that restricts gene flow between Korean coastal and offshore populations in the East Sea may be related to the complex oceanic current patterns such as eddies and upwelling, which represent impermeable barriers to population connectivity for this species. Our findings highlight that these two populations might be representative of two separate stock within Korean waters and maintain their geographically related genetic structure.

## 1 INTRODUCTION

In marine fishes, high dispersal capability and successful settlement during the early pelagic life stages provide opportunities for habitat expansion, maintenance of sustainable population sizes, and the exchange of genetic material between geographically distant populations (Jones et al., 2009; Kritzer & Sale, 2010; Strathmann et al., 2002). Maintaining relatively strong site fidelity to small home ranges is one of the most important factors in determining genetic breaks and forming population structure in reef fishes (Doherty, Planes, & Mather, 1995; Shulman & Bermingham, 1995). From the past three decades, many population genetic studies of marine fishes have demonstrated unique genetic structures may be caused by the restricted dispersal of larvae and juveniles (Froukh & Kochzius, 2007; Miller & Shanks, 2004; Roberts, 1997). Restricted dispersal between populations may be due to geographic barriers or oceanographic patterns such as ocean currents, eddies, and upwelling, which cause limited connectivity even among neighboring populations, especially during pelagic life stages (Buonaccorsi et al., 2004; Rocha‐Olivares & Vetter, 1999).

The rockfish genus *Sebastes* Cuvier, 1829 comprises approximately 110 species worldwide, despite their relatively recent divergence (Hyde & Vetter, 2007; Love, Yoklavich, & Thorsteinson, 2002; Nelson, Grande, & Wilson, 2016). Because most adult rockfishes have relatively strong site fidelity (Mitamura et al., 2002; Starr, Heine, Felton, & Cailliet, 2002), it is a taxon with high potential for forming significant within‐species population structure. Furthermore, as *Sebastes* species are ovoviviparous (i.e., the female releases free‐swimming larvae), the range and direction of transport during dispersive phases of the early life cycle are potentially important influences on population structure (Love et al., 2002). These factors, which may hinder gene flow between populations in *Sebastes* species, have the potential to promote population differentiation to occur more frequently than in other marine fish (especially pelagic fishes). Conversely, because speciation within *Sebastes* occurred fairly recently (Briggs, 1995; Hyde & Vetter, 2007), much evidence of hybridization has been reported among closely related species due to incomplete or relaxed reproductive barriers in sympatry (Artamonova et al., 2013; Muto, Kai, Noda, and Nakabo, 2013; Saha et al., 2017). This hybridization is a challenge to delimit species and populations.

The goldeye rockfish, *Sebastes thompsoni* (Jordan & Hubbs, 1925), is an important component of the commercial rockfish catch in the Northwest Pacific Ocean. In Korea, the range of this species includes the Yellow Sea, the Korea Strait, the East Sea, and around Jejudo Island (Joo, 2006; Kim & Ryu, 2016). The distribution of *S*.* thompsoni* in Japan extends southward from southern Hokkaido to Tokyo and Tsushima Island (Nakabo & Kai, 2013). *S*.* thompsoni* adults are strongly associated with deep‐water reefs (ca. 80–160 m; Kim, Han, Kang, & Kim, 2004) and exhibit unique ontogenetic behavior suitable for that period (Kokita & Omori, 1998; Nagasawa & Kobayashi, 1995). When released from brood fish (March–June, with a peak in April), the free‐swimming larvae are approximately 6 mm in body length (BL). After the planktonic larval stage (ca. 20 mm BL, 40 days), larvae and juveniles may spend up to 80 days (ca. 50 mm BL) associated with drifting seaweed, eventually settling as adults onto rocky bottom habitat (Cho, Myoung, Kim, & Hwan Lee, 2001; Kokita & Omori, 1998, 1999; Nagasawa & Kobayashi, 1995). The reproductive strategy of associating with drifting seaweed predisposes marine fishes to disperse larvae and juveniles via drifting seaweed transported by tidal, wind‐driven, and ocean currents, provides opportunities to exchange genetic material among geographically distant populations (Helmuth, Veit, & Holberton, 1994; Highsmith, 1985). Japanese halfbeak (*Hyporhamphus sajori*) and Korean rockfish (*Sebastes schlegelii*) are good examples to demonstrate this; both species spend their early life stages in drifting seaweed, showing very high genetic homogeneity despite the geographic distance between populations (Gao et al., 2016; Uchida & Shojima, 1958; Yu, Kai, & Kim, 2016).

In Japan, a previous study of *S*.* thompsoni* around the Japanese Archipelago using seven microsatellite (ms) loci found evidence of genetic homogeneity with the exception of one ms locus (Sekino, Takagi, Hara, & Takahashi, 2001). Sekino et al. (2001) asserted that the Tsushima Warm Current might be a major driver affecting genetic connectivity among local populations of Japanese *S*.* thompsoni*. Meanwhile, there are two conflicting viewpoints for the role of Tsushima Warm Current, branches from the Kuroshio Warm Current, in shaping the genetic population structure between the Korean Peninsula and Japanese Archipelago. The Tsushima Warm Current might promote the dispersal of some pelagic larvae and juveniles, and provide the opportunity for gene flow (Kasai, Komatsu, Sassa, & Konishi, 2008; Kim, Bae, Lee, & Yoon, 2017; Song, Gao, Ying, Yanagimoto, & Han, 2017). However, some previous study has shown that the disconnections among the Yellow Sea, Korea Strait, and Japanese Archipelago are related to the influence of different water masses such as the Tsushima Warm Current and Yellow Sea Bottom Cold Water (Han, Kim, Tashiro, Kai, & Yoo, 2017). Therefore, despite the previous study on Japanese *S*.* thompsoni*, further investigation of genetic diversity and connectivity among Korean *S*.* thompsoni* populations from various oceanographic perspectives is required.

*Sebastes joyneri* (Günther, 1878) is the species most closely related to *S*.* thompsoni*, from the perspectives of morphology and genetics (Hyde & Vetter, 2007; Kai, Nakayama, & Nakabo, 2003; Nakabo & Kai, 2013). This species has a greater preference for warmer habitats than *S*.* thompsoni*, despite some overlap in their distributions (Jejudo Island, Wangdolcho Reef, and Dokdo Island in Korean waters; Kim et al., 2005; Nakabo & Kai, 2013). *Sebastes joyneri* is often seen forming small groups of a few individuals, mixing with large flocks of *S*.* thompsoni* (*personal observation*). Speciation event between *S. thompsoni* and *S. joyneri* was fairly recently (Hyde & Vetter, 2007), and their present sympatry may be a secondary contact with incomplete or relaxed reproductive barriers after speciation event because their main distributional ranges are somewhat different. Furthermore, the opportunity for introgression will also increase when the spawning periods and areas of two species are in close proximity (Kijewska, Burzyński, & Wenne, 2009; Kim et al., 2017; Montanari, Van Herwerden, Pratchett, Hobbs, & Fugedi, 2012). Therefore, the introgressions occurring between close species in genus *Sebastes* may affect genetic population structure within species.

The objective of this study was therefore to clarify the connectivity among local populations of Korean *S*.* thompsoni* and its degree of hybridization with *S*.* joyneri* using the mitochondrial (mt) DNA control region and 11 polymorphic microsatellite loci.

## 2 MATERIALS AND METHODS

### 2.1 Sampling

**Figure 1:**
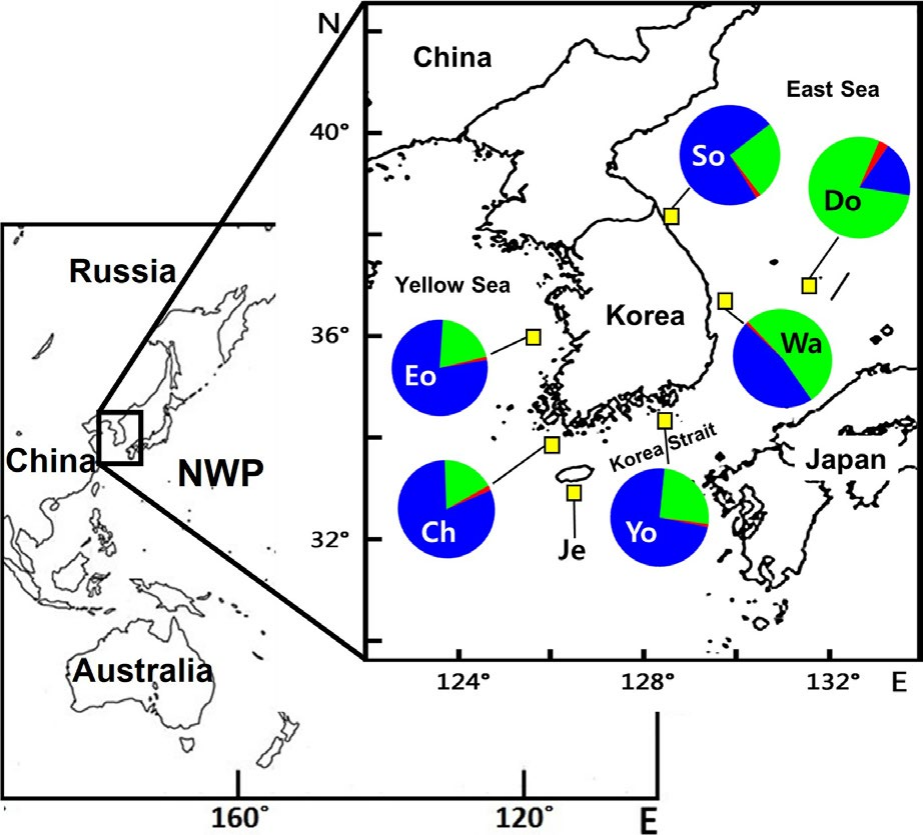
Distribution of sampling locations and geographic patterns of genetic structure for *Sebastes thompsoni*. Sampling locations are indicated by yellow rectangles with black borders. Eo: Eocheongdo Island; Ch: Chujado Island; Yo: Yokjido Island; Wa: Wangdolcho Reef; So: Sokcho; Do: Dokdo Island; and Je: Jejudo Island. Pie charts summarize the structure assignment obtained using 11 microsatellite loci, and colors indicate the proportional values of clusters inferred by STRUCTURE analysis. Red: Cluster 1; blue: Cluster 2; and green: Cluster 3 (*K* = 3, including *Sebastes joyneri*)

Samples of *S*.* thompsoni* were collected from its main habitats throughout Korean waters, from seven locations: Eocheongdo Island (Eo), Chujado Island (Ch), Yokjido Island (Yo), Wangdolcho Reef (Wa), Sokcho (So), Dokdo Island (Do), and Jejudo Island (Je); *S*.* thompsoni* appeared infrequently in Sokcho (Figure 1). We used 91 tissue samples of *S. thompsoni* from seven locations for the mtDNA sequence analysis. We also used 215 tissue samples of *S. thompsoni* from six locations (Jejudo Island was excluded due to small sample size) for the ms loci analysis, in which we analyzed at least 30 individuals per location. Samples of fish from five sampling locations (Ch, Je, Yo, Wa, and So) were collected during 2012–2016 by commercial fishing vessels, and samples of fish from Dokdo (Do) were collected by commercial fishing vessels and research surveys (most samples) during 2011–2014. In the remaining sampling location (Eo), samples were directly collected by fishing from a boat in December 2013 and October 2014. We also included *S*.* joyneri* samples to examine its genetic relationship with *S*.* thompsoni*. Samples of *S*.* joyneri* were collected during 2010–2016 from three locations (Je, Wa, and Do) by commercial fishing vessels, with habitats overlapping those of *S*.* thompsoni* in Korean waters. We used 29 tissue samples of *S. joyneri* for the mtDNA sequence analysis and 48 tissue samples of *S. joyneri* for the ms loci analysis.

Sample identification was conducted according to the morphological characteristics described by Kim et al. (2005) and Nakabo and Kai (2013). Species abbreviations were chosen using the initials of their scientific names (e.g., *S*.* thompsoni*: ST; *S*.* joyneri*: SJ) and the first letters of the sampling location (e.g., Eocheongdo Island: Eo), joined by an underscore. The tissue samples were preserved in 99% ethanol and stored at −20°C until DNA extraction. The specimens used in this study were deposited at Pukyong National University (PKU).

### 2.2 Laboratory protocols

DNA was extracted from muscle tissue using 10% Chelex 100 Resin (Bio‐Rad; http://www.bio-rad.com) according to the manufacturer's instructions. The region between the proline transfer RNA (tRNA^Pro^) gene and the end of the control region in the mtDNA gene was amplified using the primer set L15876 (5′‐AAGCACTTGAATGAGCTTG‐3′) (Rocha‐Olivares & Vetter, 1999) and H851219 (5′‐GTTTTCCTGTTTCCGGGGGGTTT‐3′), designed specifically for species of the genus *Sebastes*, by PCR. PCR was performed in an MJ Mini 48‐Well Thermal Cycler (Bio‐Rad) with a reaction mixture containing 1 μl of total DNA, 2 μl of 10× PCR buffer, 1.6 μl of 2.5 mmol/l dNTPs, 0.5 μl of each primer, 0.1 μl of Ex Taq DNA Polymerase (TaKaRa Bio Inc.; http://www.takara-bio.com), and distilled water to a final volume of 20 μl. The cycling conditions were an initial denaturation step for 1 min at 96°C followed by 35 cycles of 1 min at 96°C, 1 min at 56°C, and 2 min at 72°C, with a final step of 10 min at 72°C. The PCR products were purified using a LaboPass PCR Purification Kit (Cosmogenetech Co.; http://www.cosmogenetech.com). The mtDNA was sequenced on an ABI 3730XL Sequencer (Applied Biosystems; http://www.appliedbiosystems.com) using an ABI BigDye Terminator Cycle Sequencing Ready Reaction Kit v3.1 (Applied Biosystems).

We also selected 11 microsatellite markers from the loci designed for *S*.* thompsoni*: Sth3A, Sth3B, Sth24, Sth37, Sth45, Sth56, Sth86, and Sth91 (Sekino, Takagi, Hara, & Takahashi, 2001) and *S*.* schlegelii*: KSs2A, KSs3, and KSs27A (An, Park, Kim, Lee, & Kim, 2009). All markers were labeled with FAM, HEX, and TAMRA fluorescent dyes for the forward primer. Multiplex PCR amplification of nine markers was performed in three sets, and singleplex PCR was also performed for two markers (Sth37 and KSs3) due to limitations of conditional matching. The protocol followed was that outlined by Sekino et al. (2001) and An et al. (2009) for the PCRs, fragment amplification, and scoring the microsatellite loci. The multiplex and single PCR products were combined and analyzed on an ABI 3730XL Sequencer (Applied Biosystems) using the GeneScan 500 LIZ dye size standard (Macrogen Inc.; http://www.macrogen.com). The alleles were scored with GeneMapper 3.7 (Applied Biosystems).

### 2.3 Data analysis

Because the analyzed sample size in the mtDNA control region was relatively small, the analysis included only an examination of the basic molecular characteristics and phylogenetic relationships of both species. The mtDNA sequences were aligned using ClustalW (Thompson, Higgins, & Gibson, 1994) in BioEdit 7 (Hall, 1999). Haplotype (h) and nucleotide (π) diversities were estimated according to Nei (1987) in Arlequin 3.5.1.2 (Excoffier, Laval, & Schneider, 2005). The phylogenetic trees for mtDNA sequencing data were obtained using the neighbor‐joining (NJ) method (Saitou & Nei, 1987) with 1000 bootstrap replicates and Tamura–Nei model distances (Tamura & Nei, 1993), which are appropriate to describe the evolution of mtDNA control region sequences, using the MEGA 6 software (Tamura, Stecher, Peterson, Filipski, & Kumar, 2013). The genetic distance (*d*) between and within species was calculated using the Kimura two‐parameter model (Kimura, 1980) as implemented in the MEGA software (Tamura et al., 2013). To estimate the genetic divergence (*d*) among *S*.* thompsoni* sampling locations, the pairwise *F*_ST_ values were calculated with 10,000 permutations using Arlequin 3.5.1.2 (Weir & Cockerham, 1984).

In the ms marker data analysis, the average number of alleles per locus (*N*a), allelic richness (Ar), and inbreeding coefficient (*F*_IS_) were calculated using FSTAT 2.9.3.2 (Goudet, 1995). Micro‐Checker v2.2 (Van Oosterhout, Hutchinson, Wills, & Shipley, 2004) was used to detect the presence of null alleles and genotyping errors such as stuttering and large allele dropout. Deviations from Hardy–Weinberg equilibrium (HWE) for each microsatellite locus were estimated using Genepop 3.1 (Rousset, 2008) and *p* values estimated using the Markov chain Monte‐Carlo method (MCMC; 10,000 dememorization steps, 1000 batches, and 10,000 iterations). Observed (*H*_O_) and expected (*H*_E_) heterozygosities were estimated using Arlequin 3.5.1.2 (Excoffier et al., 2005). Polymorphic information content (PIC) was calculated using Cervus 3.0.6 (Kalinowski, Taper, & Marshall, 2007). Pairwise *F*_ST_ values between sampling locations within species or between *S*. *thompsoni* and *S*. *joyneri* were estimated with 10,000 permutations using Arlequin 3.5.1.2 (Weir & Cockerham, 1984). Significance levels for multiple tests were adjusted by the sequential Bonferroni correction (0.05/11C2 = 0.0009; *p* < .0009). The model‐based Bayesian clustering procedure in STRUCTURE 2.3.4 (Pritchard, Stephens, & Donnelly, 2000) was used to determine how many genetic groups (*K*) best represented the study areas. In this analysis, an admixture model without prior sampling location information was used and allowed for correlations between allele frequencies (Falush, Stephens, & Pritchard, 2003). Posterior probabilities were generated for *K* values 1–9 using 1,000,000 iterations and the MCMC method, with a burn‐in of 500,000 iterations, to calculate the probable *K* value. STRUCTURE HARVESTER 0.6.94 (Earl & vonHoldt, 2012) was used to apply the Evanno method (Evanno, Regnaut, & Goudet, 2005) to determine the value of *K* that was appropriate for a genetic cluster. Individuals used in STRUCTURE analysis were identified as possible hybrids if at least 10% of their genome (*q*) originated from other groups (Randi, 2008). Discriminant analysis of principal components (DAPC; Jombart, Devillard, & Balloux, 2010) was used to identify clusters based on the spatial distribution of microsatellite genotypes, using the *adegenet* 2.0.1 package in R 3.2.2 (http://www.r-project.org; Jombart, 2008). Initially, genetic data were transformed into uncorrelated components using principal component analysis (PCA), and principal components (PC) were then submitted to discriminant analysis (DA). In this study, 50 PCs were retained, containing 80% of the variation in the data.

Analysis of molecular variance (AMOVA) was performed to test the best hierarchical groupings based on two alternative hypotheses in sampling areas using Arlequin 3.5.1.2 (Excoffier, Smouse, & Quattro, 1992). The first hypothesis is that Korean *S. thompsoni* populations in sampling areas form only one cluster. The second hypothesis is that Korean *S. thompsoni* populations are divided into two clusters (Eo, Ch, Yo, So, and Wa‐1 vs. Wa‐2, Do) in sampling areas. Here, we separated the Wangdolcho Reef population of *S*. *thompsoni* into two genetic clusters (Wa‐1 and Wa‐2); this decision followed criteria for the majority cluster (Cluster 2 or 3) in the STRUCTURE analysis (*K* = 3). The best grouping was defined by the highest differentiation among groups (*F*_CT_) and nonsignificant differentiation within groups (*F*_SC_). *F*‐statistics were obtained in each analysis from 10,000 permutations in Arlequin 3.5.1.2 (Excoffier et al., 1992). Finally, isolation‐by‐distance (IBD) analysis was performed using a pairwise *F*_ST_ matrix, with a Mantel test between populations in IBDWS (http://ibdws.sdsu.edu/~ibdws; Bohonak, 2002; Jensen, Bohonak, & Kelley, 2005), to test for correlation between genetic and geographic distances.

## 3 RESULTS

### 3.1 Genetic diversity

In total, 765 base pairs (bp) of the mtDNA control region sequences were amplified in 91 *S*.* thompsoni* individuals (seven sampling locations) and 29 *S*.* joyneri* individuals (three sampling locations) collected in Korean waters. In *S*. *thompsoni*, 189 polymorphic sites were detected, with 183 transitions and 21 transversions. We identified 90 haplotypes from 91 individuals and detected very high haplotype (*h *=* *0.9998) and nucleotide (π = 0.0231) diversity (Table 1). The genetic distance (*d*) within *S. thompsoni* ranged from 0.000 to 0.054 (average 0.024), indicating a relatively high intraspecific genetic discrepancy. In *S*.* joyneri*, 61 polymorphic sites were detected, with 56 transitions and five transversions. We identified 25 haplotypes from 29 individuals, and detected relatively high haplotype (*h *=* *0.9901) and nucleotide (π = 0.0125) diversity, although somewhat lower than that of *S*.* thompsoni* (Table 1). Genetic distance (*d*) compared between *S*.* joyneri* individuals ranged from 0.000 to 0.026, with an average distance among all individuals of 0.013.

**Table 1:** Summary of molecular diversity, mutation neutrality, and statistics across mtDNA control region (765 bp) and 11 microsatellite loci for six *S*.* thompsoni* and three *S*.* joyneri* populations from each sampling location in Korean waters

Species	Sampling location	Abbreviation	Collection date	*n*	*N*	*h*	π	*n*’	*N*_A_	Ar	*H*_E_	*H*_O_	PIC	*F*_IS_
*S*.* thompsoni*	Eocheongdo Island (Eo)	ST_Eo	2013.12/2014.01/2014.10	20	20	1.0000	0.0226	42	8.36	7.09	0.6509	0.5573	0.6115	0.145
	Chujado Island (Ch)	ST_Ch	2014.01	10	10	1.0000	0.0126	33	8.18	7.38	0.6555	0.6384	0.6162	0.026
	Yokjido Island (Yo)	ST_Yo	2014.01	11	11	1.0000	0.0130	32	8.00	7.22	0.6494	0.6148	0.6093	0.054
	Sokcho (So)	ST_So	2008.11–2014.01	12	12	1.0000	0.0223	30	8.27	7.42	0.6753	0.6495	0.6333	0.039
	Wangdolcho Reef (Wa)	ST_Wa	2010.05/2014.03/2016.08	16	16	1.0000	0.0261	37	8.91	7.58	0.7031	0.6347	0.6580	0.099
	Dokdo Island (Do)	ST_Do	2011.02/2011.03/2012.02/2014.07	15	15	1.0000	0.0210	41	9.82	7.89	0.6884	0.5944	0.6499	0.138
	Jejudo Island (Je)	SJ_Je	2011.4/2013.7	7	7	1.0000	0.0216	–	–	–	–	–	–	–
	Combined			91	90	0.9998	0.0231	215	8.59	7.43	0.6704	0.6149	0.6297	0.100
*S*.* joyneri*	Wangdolcho Reef (Wa)	SJ_Wa	2016.08	7	7	1.0000	0.0127	13	6.36	7.32	0.6929	0.6453	0.7652	0.071
	Jejudo Island (Je)	SJ_Je	2011.02/2011.05/2011.08/2011.11	13	13	1.0000	0.012	17	7.10	6.93	0.6897	0.6382	0.7554	0.077
	Dokdo Island (Do)	SJ_Do	2010.05/2011.04/2013.04/2016.08	9	9	1.0000	0.0140	18	7.73	7.73	0.6528	0.5811	0.7673	0.113
	Combined			29	25	0.9901	0.0125	48	6.88	7.33	0.6785	0.6215	0.7626	0.087

*n*: number of specimens (mtDNA control region); *N*: number of haplotypes; *h*: haplotype diversity; π: nucleotide diversity; *n*’: number of specimens (microsatellite DNA); *N*_A_: average number of alleles per locus; Ar: average allelic richness; *H*_O_: observed average heterozygosity; *H*_E_, expected average heterozygosity; PIC, polymorphic information content; and *F*_IS_, average inbreeding coefficient values.

In the microsatellite loci analysis, 215 *S*.* thompsoni* individuals (six sampling locations) and 48 *S*.* joyneri* individuals (three sampling locations) collected in Korean waters were screened using 11 microsatellite loci (Table 1). In *S*.* thompsoni*, the average number of alleles per locus was 8.59, with a range of 8.00 (Yokjido Island) to 9.82 (Dokdo Island). The average allelic richness (Ar) among all individuals was 7.43, with a range of 7.09 (Eocheongdo Island) to 7.89 (Dokdo Island). The expected and observed average heterozygosities of each sampling location among all loci ranged from 0.6494 (Yokjido Island) to 0.7031 (Wangdolcho Reef) and from 0.5573 (Eocheongdo Island) to 0.6495 (Sokcho Island); the average expected and observed heterozygosities overall were 0.6704 and 0.6149, respectively. Significant (*p* < .05) departures from HWE were found at 18 independent loci (Sth37 at ST_Eo; Sth3B at ST_Eo; Sth24 at ST_Do; Sth3A at ST_Wa; Sth56 at ST_Eo, ST_Ch, ST_Yo, ST_Wa, and ST_So; Sth91 at ST_Eo, ST_Yo, ST_Wa, ST_So, and ST_Do; KSs2A at ST_Eo; and KSs3 at ST_Eo, ST_Ch, and ST_Wa). After applying the Bonferroni correction for multiple comparisons, however, significant (*p* < .0009) departures from HWE were confirmed for only five independent loci (KSs3 at ST_Eo; Sth56 at ST_Wa; and Sth91 at ST_Yo, ST_So, and ST_Do). This deviation from HWE was observed with the presence of null alleles in the microchecker analysis, while no evidence of large allele dropout or scoring errors due to stuttering were shown. Some ms loci (Sth56, Sth91, KSs2A, and KSs3) showed significant differentiation of allele frequencies among sampling locations. In particular, some alleles in the Dokdo Island population showed a much higher (Sth56^188^, Sth91^211^, KSs2A^144^, and KSs3^145^) or lower (Sth91^213^) frequency than observed in populations at other sampling locations or did not show up (KSs2A^146^; see Figure S1). In *S*.* joyneri*, the average number of alleles per locus was 6.88, and the average expected and observed heterozygosities among all sampling locations and loci were 0.6785 and 0.6215, respectively (Table 1). Average allelic richness among all individuals was 7.33. Significant (*p* < .05) departures from HWE were found at some independent loci (Sth37 at SJ_Wa; Sth56 at SJ_Wa, SJ_Je, and SJ_Do; Sth91 at SJ_Wa and SJ_Do; and KSs3 at SJ_Je and SJ_Do). However, only two independent loci (Sth91 at SJ_Wa; and KSs3 at SJ_Je) exhibited significant (*p* < .0009) departures from HWE after the Bonferroni correction was applied.

### 3.2 Introgressive hybridization between *Sebastes* species

In the NJ tree based on the mtDNA control region sequences, *S*.* thompsoni* and *S*.* joyneri* were clearly distinguished, except for one individual (PKU_5153, collected from Dokdo Island) that identified morphologically as *S*.* thompsoni* but was genetically similar to *S*.* joyneri*. The average genetic distance (*d*) between *S*.* thompsoni* and *S*.* joyneri* was 0.047 (Figure S2).

**Figure 2:**
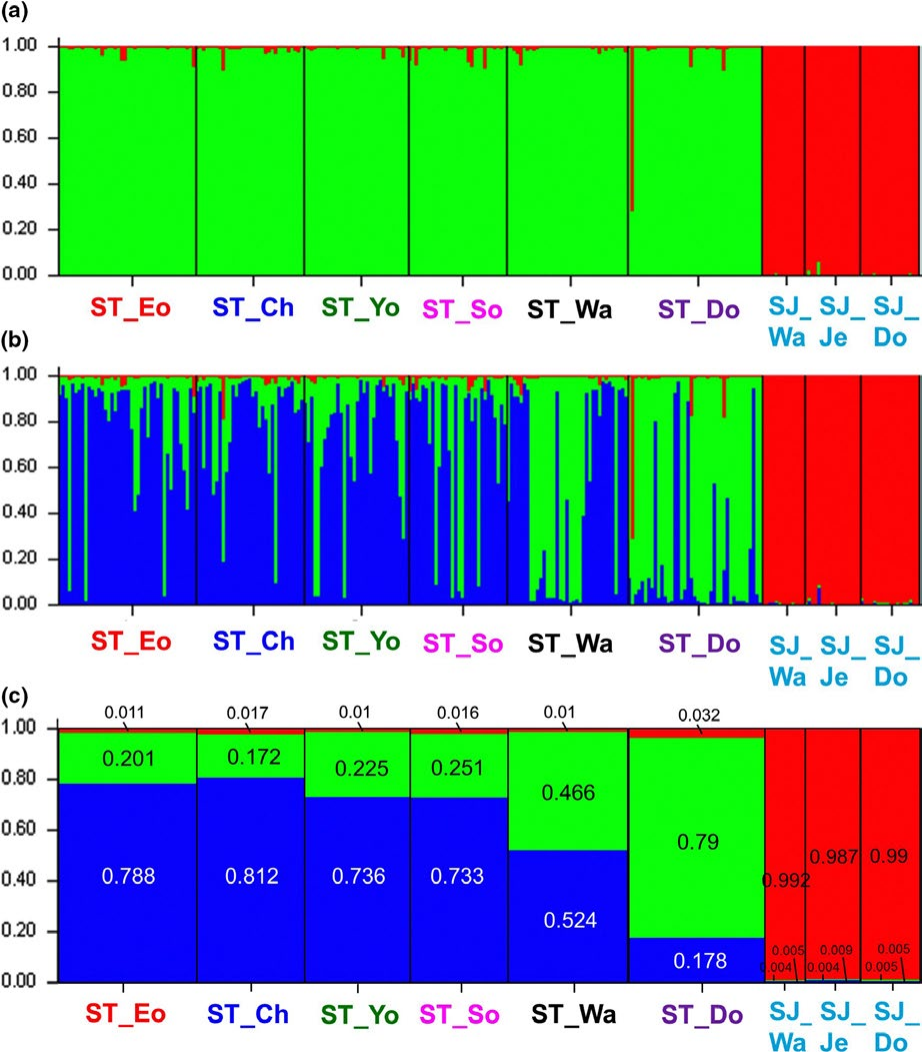
Population structure genetically determined for *Sebastes thompsoni* (ST) and *Sebastes joyneri* (SJ) from Korean waters, inferred using the STRUCTURE program. Results are shown for (a) *K* = 2 and (b) *K* = 3. Proportional values are shown for each cluster within a sampling location when (c) *K* = 3. Each color represents a genetic group defined by the STRUCTURE analysis. Abbreviations for sampling locations are defined in Figure 1 and Table 1

According to the STRUCTURE analysis based on 11 ms loci, when *K* = 2, all *S*.* joyneri* individuals were clearly separated from those of *S*.* thompsoni*, which comprised two genetically different compositions (Figure 2a). When *K* is 3 in STRUCTURE analysis, four *S*.* thompsoni* individuals (including PKU_5153), collected in the Chujado and Dokdo Islands, were detected as hybrids between *S*.* thompsoni* and *S*.* joyneri* according to the admixture proportion (.1 < *q *<* *.9) (Figure 2b, Table 2). However, except for single individual PKU_5153, the remaining three putative hybrid individuals cannot be recognized as hybridization because they did not share a rare allele derived from *S. joyneri*.

**Table 2:** Purebred and admixture proportions among clusters for six *S*.* thompsoni* populations as determined by STRUCTURE analysis

Sampling locations	*n*	Purebred genotypes			Intraspecific hybrid		Interspecific hybrid	
		Cluster 1	Cluster 2	Cluster 3	*N* of genotype	%	*N* of genotype	%
Eocheongdo Island (Eo)	42	0	25	3	14	33.3	0	0.0
Chujado Island (Ch)	33	0	18	0	14	42.4	1	3.0
Yokjido Island (Yo)	32	0	12	3	17	53.1	0	0.0
Sokcho (So)	30	0	15	5	10	33.3	0	0.0
Wangdolcho Reef (Wa)	37	0	16	13	8	21.6	0	0.0
Dokdo Island (Do)	41	0	3	26	9	21.9	3	7.3
Combined	215	0	89	50	72	34.1	4	1.7

Purebred genotypes = *q *≥* *.90; hybrid genotypes = 0.1 < *q *<* *.9. Intraspecific hybrid: hybrid genotypes between Clusters 2 and 3; interspecific hybrid: hybrid genotypes between Clusters 1 and 2 or 3. *n*: number of specimens (mtDNA control region); and *N*: number of haplotypes.

### 3.3 Genetic population structure

The NJ tree of *S*.* thompsoni* populations based on the mtDNA control region sequences showed a single panmictic group (Figure S2), supported by no significant differences of pairwise *F*_ST_ values (*p* > .05). Hence, we found no evidence of genetic population structure even when using hypervariable mtDNA control region sequences. In contrast to the mtDNA analysis results, two clusters were found in the Korean *S*.* thompsoni* populations based on ms loci. Pairwise *F*_ST_ comparisons of the Dokdo Island population with other local populations indicated highly significant differences after the Bonferroni correction was applied (*p* < .0009), with a range of 0.0175 (Wangdolcho Reef) to 0.0639 (Chujado Island; Table 3). The Wangdolcho Reef population also showed a significant difference (*p* < .05) in pairwise *F*_ST_ values with Eocheongdo (0.0145), Chujado (0.0236), Yokjido (0.0194), and Dokdo (0.0175) Island populations; however, after the Bonferroni correction was applied (*p* < .0009), the pairwise *F*_ST_ values were only significant for the Chujado, Yokjido, and Dokdo Island populations. Other evidence for population genetic differentiation within *S*.* thompsoni* was detected by the STRUCTURE analysis (Figure 2). In this study, because the *F*_ST_ values calculated between populations from Korean *S. thompsoni* showed a significant difference, the genetic divergence among populations may hint that there is indeed structure. Therefore, although the largest value of *K* in the STRUCTURE HARVESTER analysis occurred at *K* = 2 (*S*. *thompsoni* vs. *S*.* joyneri*), reflecting large genetic differences between species (*F*_ST_ = 0.2181, *p* = .0000), the next highest *K* value (*K* = 3) was adopted as the most suitable number of clusters. For this reason, when *K* is 3, STRUCTURE analysis may allow to detect at lower levels of genetic variation inferred from the Korean *S. thompsoni* populations.

**Table 3:** Pairwise *F*_ST_ values for microsatellite loci of six *S*.* thompsoni* (ST) populations and three *S*.* joyneri* (SJ) populations from Korea. Abbreviations for geographic locations are defined in Figure 1 and Table 1

	ST_Eo	ST_Ch	ST_Yo	ST_So	ST_Wa	ST_Do	SJ_Wa	SJ_Je	SJ_Do
ST_Eo									
ST_Ch	−0.0018								
ST_Yo	0.0031	0.0060							
ST_So	−0.0024	0.0027	0.0049						
ST_Wa	**0.0145**	**0.0236** a	**0.0194** a	0.0054					
ST_Do	**0.0573** a	**0.0639** a	**0.0542** a	**0.0411** a	**0.0175** a				
SJ_Wa	**0.2216** a	**0.2189** a	**0.2339** a	**0.1925** a	**0.2141** a	**0.2368** a			
SJ_Je	**0.2316** a	**0.2321** a	**0.2487** a	**0.2158** a	**0.2362** a	**0.2686** a	**0.0235**		
SJ_Do	**0.2206** a	**0.2222** a	**0.2368** a	**0.1982** a	**0.2256** a	**0.2509** a	0.0072	0.0061	

Bold values showed significant differences (*p* < .05).

a Differences were significant after Bonferroni correction (*p* < .0009).

**Figure 3:**
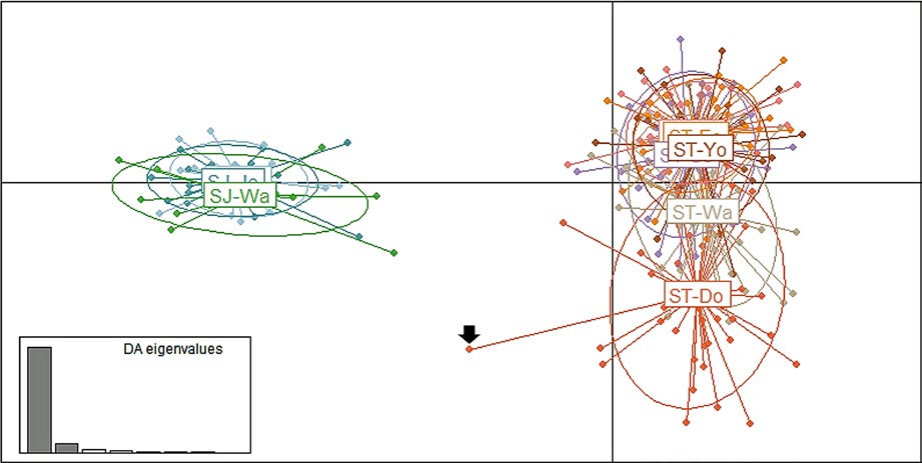
Scatter plots of the discriminant analysis of principal components (DAPC) of the microsatellite data for six *Sebastes thompsoni* (ST) populations and three *Sebastes joyneri* (SJ) populations from Korean waters. Individual genotypes appear as dots surrounded by 95% inertia ellipses. Arrows indicate individuals showing possible hybridization between ST and SJ. Abbreviations for geographic locations are defined in Figure 1 and Table 1

The STRUCTURE analysis obtained with *K* = 3, including *S*.* joyneri* populations (Cluster 1), confirmed the genetic divergence between two clusters (Clusters 2 and 3) within the *S*.* thompsoni* populations, separated at the boundary of Wangdolcho Reef (Figure 2b). As a proportional values represented by each cluster within sampling areas, the Eocheongdo Island (0.788 in Cluster 2 vs. 0.201 in Cluster 3), Chujado Island (0.812 vs. 0.172), Yokjido Island (0.736 vs. 0.255), and Sokcho (0.733 vs. 0.251) populations had a much higher proportion of Cluster 2, whereas the Dokdo Island (0.178 vs. 0.790) population had a much higher proportion of Cluster 3 (Figure 2c). The purebred individuals identified in the STRUCTURE analysis also exhibited similar clustering patterns like the proportional values of each cluster (Table 2). The Wangdolcho Reef population made up a similar proportion of two clusters (0.524 in Cluster 2 vs. 0.466 in Cluster 3) represented by each cluster as well as purebred individuals (16 individuals in Cluster 2 vs. 13 individuals in Cluster 3), forming approximately half of the cluster proportions, respectively (Figure 2c, Table 2). The first variance component (*x*‐axis) on the DAPC plot separated the Dokdo population from other sampling locations (except the Wangdolcho Reef population), although some individuals that were identified as Cluster 2 in the STRUCTURE analysis overlapped (Figure 3). The Wangdolcho Reef population was also located in the middle of the two subpopulations in the DAPC plot, suggesting that the two‐cluster individuals are mixed.

**Figure 4:**
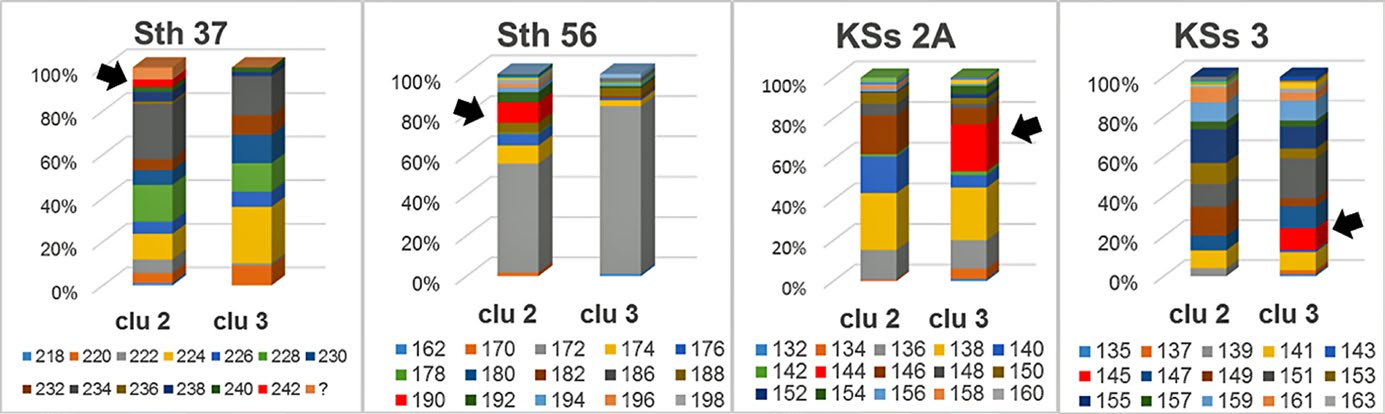
Allele frequency distributions for purebred individuals of two clusters (clu) in *Sebastes thompsoni* populations at six sampling locations, based on four microsatellite loci showing differences between populations. Arrow indicates a unique allele detected in only one cluster

Finally, allele frequencies appeared different between purebred individuals represented by each cluster regardless of sampling location, and a few unique alleles (Sth37^242^, Sth56^190^, KSs2A^144^, and KSs3^145^) observed in only one cluster were also detected in some ms loci (Figure 4).

The results of AMOVA showed that the two *S*.* thompsoni* groupings, as well as one *S*.* joyneri* grouping (SJ_Wa, SJ_Je, and SJ_Do), were significantly supported, where the ST_Eo, ST_Ch, ST_Yo, ST_So, and ST_Wa‐1 samples were observed as separate genetic groups from the ST_Wa‐2 and ST_Do samples, according to the highest *F*_CT_ values (*F*_CT_ = 0.1543, *p* < .009) and lower or nonsignificant *F*_SC_ values (*F*_SC_ = 0.0058, *p* > .009) using ms marker data (Table 4). Finally, there was no indication of genetic isolation by distance between the geographic ranges of individuals collected (Figure S3).

**Table 4:** Results of analyses of molecular variance (AMOVAs) using microsatellite data for *S*.* thompsoni* and *S*.* joyneri* from Korea

Number of clusters within *S*. *thompsoni*	Configuration		11 microsatellite loci		
			*F*_ST_	*F*_SC_	*F*_CT_
1	(ST_Eo, ST_Ch, ST_Yo, ST_So, ST_Wa‐1, ST_Wa‐2, ST_Do) vs. (SJ_Wa, SJ_Je, SJ_Do)	Fixation indices	**0.2352**	**0.0281**	0.2131
		*p* value	.0000	.0000	.0094
2	(ST_Eo, ST_Ch, ST_Yo, ST_So, ST_Wa‐1) vs. (ST_Wa‐2, ST_Do) vs. (SJ_Wa, SJ_Je, SJ_Do)	Fixation indices	**0.1592**	0.0058	**0.1543**
		*p* value	.0000	.0081	.0008

ST: *Sebastes thompsoni*; SJ: *Sebastes joyneri*; Eo: Eocheongdo Island; Ch: Chujado Island; Yo: Yokjido Island; So: Sokcho; Wa: all individuals from Wangdolcho Reef; Wa‐1: two‐cluster (STRUCTURE analysis, *K* = 3) individuals from Wangdolcho Reef; Wa‐2: 3‐cluster (STRUCTURE analysis, *K* = 3) individuals from Wangdolcho Reef; Do: Dokdo Island; and Je: Jejudo Island. Genetic variation was partitioned as: *F*_ST_ = within populations, *F*_SC_ = among populations within groups, and *F*_CT_ = among groups.

STRUCTURE analysis revealed admixed genotypes in individuals (.1 < *q* < .9) between Clusters 2 and 3 in *S*.* thompsoni* (Figure 2b). In fact, the highest proportion of intraspecific hybrids (hybrid genotypes between Clusters 2 and 3) was found in Yokjido Island (53.1%), followed by Chujado Island (42.4%), Eocheongdo Island (33.3%), Sokcho (33.3%), Dokdo Island (21.9%), and Wangdolcho Reef (21.6%; Table 2).

Additionally, for *S*.* joyneri* individuals collected from three locations in Korean waters, no significant difference was found in *F*_ST_ values of mtDNA (*p* > .05) and msDNA (*p* > .0009) data (Table 3), and there was no evidence of genetic population structure identified with the STRUCTURE or DAPC analysis using msDNA data (Figures 2 and 3).

## 4 DISCUSSION

The present study found discordance between the mtDNA and msDNA loci results; the former suggested that Korean *S*.* thompsoni* comprised a single panmictic group, but the latter suggested that it comprised two genetically distinct clusters: Cluster 2 was predominant in Korean coastal locations (the Eocheongdo, Chujado, and Yokjido Islands, and Sokcho); Cluster 3 was predominant at Dokdo Island; and both clusters occurred in similar ratios at Wangdolcho Reef. The subdivision of the two clusters in Korean *S*.* thompsoni*, unlike Japanese *S*.* thompsoni*, may be due to the restricted dispersal of larvae and juveniles by complex oceanographic patterns in the East Sea. Furthermore, a relatively high admixture proportion between the two clusters in most sampling locations, in particular Chujado and Yokjido Island, has been identified, possibly due to disassortative mating associated with bias in occurrence ratios in each cluster.

A deficiency of heterozygotes and the positive value of *F*_IS_ of Korean *S*.* thompsoni* in this study reflect a significant deviations from HWE owing to a difference in null allele frequencies between samples. Moreover, the frequency of null allele ranges from 0.05 to 0.33, suggesting the deviation from HWE could be associated with the presence of null alleles. On the other hand, it does not seem to be a Wahlund effect because of no high migration and no IBD structure in this species. The presence of null alleles, which caused an excess of homozygotes, can overestimate the levels of genetic differentiation (Chapuis & Estoup, 2006). However, many studies demonstrated that the analysis conducted excluding the loci with null alleles produced very similar results of genetic differentiation as when they were included (Dick, Shurin, & Taylor, 2014; Lane, Symonds, & Ritchie, 2016; Underwood, Travers, & Gilmour, 2012). Furthermore, it is known that null alleles could not change the overall patterns of genetic population revealed in ms loci analysis (Carlsson, 2008).

High levels of genetic diversity (overall *h *=* *0.9998, π = 0.0231) of mtDNA control region of Korean *S. thompsoni* were detected in this study, which is higher than other *Sebastes* species (*S. zonatus* [*h *=* *0.978, π = 0.014; Muto et al., 2013]; *S. vulpes* [*h *=* *0.937, π = 0.014; Muto et al., 2013]; *S. longispinis* [*h *=* *0.96, π = 0.0070; Kai, Park, & Nakabo, 2012]; *S. hubbsi* [*h *=* *0.97, π = 0.0068; Kai et al., 2012]; *S. inermis* complex [*h *=* *0.98, π = 0.013; Kai, Nakayama, & Nakabo, 2002]; *S. schlegelii* [*h *=* *0.46, π = 0.0019; Higuchi & Kato, 2002]; and *S. owstoni* [*h *=* *0.69, π = 0.0046; Higuchi & Kato, 2002]) distributed from the Northwest Pacific Ocean. In addition, the nucleotide diversity (overall π = 0.0231) of Korean *S. thompsoni* was over two times higher than the average value of nucleotide diversity (π = 0.0104) from 11 *Sebastes* species distributed in the Northeast Pacific Ocean (Hess et al., 2013). Therefore, high levels of haplotype and nucleotide diversity indicated that Korean *S. thompsoni* populations would have suffered large stable population with long evolutionary history or secondary contact between differentiated lineages (Grant & Bowen, 1998). However, reduced levels of genetic diversity in mtDNA and heterozygous deficiency in ms loci were remarkably observed in Chujado (π = 0.0126, *H*_E_ = 0.6555) and Yokjido (π = 0.0130, *H*_E_ = 0.6494) Island populations. There are several possible explanations for such a finding: small sample size, selection, immigration, or genetic drift. Although the sample size used for analysis in all populations is similar or slightly different, low genetic diversity was confirmed only in Chujado and Yokjido Island populations. It is also unlikely that effect of selection or immigration occurred at Chujado and Yokjido Island in Korea Strait because of their main stable habitat. Thus, genetic drift within the both sites is the most likely explanation for relative low genetic diversity, implying a reduction in effective population size due to local overfishing (Frankham, Ballou, & Briscoe, 2010). Actually, most fishery for this species is concentrated in Korea Strait, including Chujado and Yokjido Island, for decades due to the relatively low depth of water and the ease of accessibility, so it has been exposed to the risk of local overfishing. The effects of local overfishing on genetic diversity have been demonstrated at the population study of New Zealand snapper (*Pagrus auratus*) that showed the significant decrease in both heterozygosity and the mean number of alleles in one population (Tasman Bay) during its exploitation history (Hauser, Adcock, Smith, Ramírez, & Carvalho, 2002). Similarly, in this study, these declines were also observed in the Chujado and Yokjido Island populations (Table 1). Therefore, Korean *S. thompsoni* showed a high genetic diversity, but the difference of diversity among sampling locations has been identified due to human activity.

According to Muto et al. (2013), interspecific hybridization between *Sebastes vulpes* and *Sebastes zonatus*, occurring in the Northwest Pacific Ocean, was detected in a total of 63 (35.6%) individuals using a combination of amplified fragment length polymorphisms (AFLP), mtDNA, and morphometric characters. Interestingly, this study showed that rates of hybridization varied considerably among the locations, which is associated with variations in habitat segregation, implying that the extent of habitat segregation of the two species dependent on vertical water temperature regimes determined the opportunity for hybridization. Four *Sebastes* species in the North Atlantic Ocean have also shown evidence of interspecific hybridization when in sympatry (Artamonova et al., 2013; Pampoulie & Daníelsdóttir, 2008; Roques, Sevigny, & Bernatchez, 2001). Moreover, Saha et al. (2017) suggested that interspecific introgression might influence allele frequency differences among *S*.* mentella* populations in sympatry. In case of *S. thompsoni* and *S. joyneri* in Korean waters, the depth of water at which both species are caught off Jejudo Island, Wangdolcho Reef, and Dokdo Island is also similar, indicating a high potential for hybridization/introgression between the two species (*Personal observation*). Nevertheless, we found evidence of interspecific hybridization between Korean *S. thompsoni* and *S. joyneri* in only one individuals (PKU_5153, mtDNA and ms loci results), although there was suggestion that the possibility of the hybridization of four individuals in the STRUCTURE analysis. Only one individual identified as hybrid was not only at least 10% of the genome (*q*) originating from another cluster in the STRUCTURE analysis (Randi, 2008) but also shares the pure both parental genotypes (*q *≥* *.90). The occurrence ratio of hybridization between the two species was considerably lower than expected, despite the high likelihood of hybridization due to extensive overlap in the mating and release seasons of the two species (Kokita & Omori, 1998; Nagasawa & Kobayashi, 1995; Yang et al., 2016). Consequently, in this study, the observed low hybridization rates between Korean *S*.* thompsoni* and *S*.* joyneri* suggest that there was no effect on the determination of population structure of Korean *S*.* thompsoni*.

Korean *S*.* thompsoni* was found to be genetically indistinguishable between sampling locations using the mtDNA control region sequence. Previous study in the Japanese Archipelago, which was performed preliminarily for 10 individuals in only two regions (East Sea and Pacific coast), respectively, suggest that genetic population structure is likely a single panmictic group like this study, but showed a higher genetic diversity (overall *h *=* *1.0000, π = 0.0345) rather than Korean *S. thompsoni* (overall *h *=* *0.9998, π = 0.0231; Higuchi & Kato, 2002). This would suggest that stable population levels and extensive gene flow in Japan have fostered the generation and persistence of very high genetic diversity (Grant & Bowen, 1998). However, as only two locations and a few individuals in Japan were analyzed in the previous study, further studies of various locations and many samples should be carried out to corroborate this finding. In the Northwest Pacific Ocean, the isolation of the East Sea from the East China Sea during low sea levels is associated with glaciation, which would have blocked the supply of warm currents (Tsushima Warm Current) from the Korea Strait. Such an event would interrupt the connectivity of the two oceanic populations, exposing the East Sea population to unfavorable environmental conditions and causing a sequence of extinction events (Ujiié & Ujiié, 1999; Hu et al., 2011; Im, Jo, Ji, Myoung, & Kim, 2017). Therefore, during a period of frequent glacial–interglacial cycles, Korean *S*.* thompsoni* might have undergone repetitive recolonization events, with the segregation of the southern Korean population in refugia and local extinction of the East Sea population. The sea level rise in the East Sea may have caused a dramatic increase in suitable habitats for *S*.* thompsoni*; for example, the flat top of the Wangdolcho Reef may now be located closer to the sea surface than during the glacial period. A similar result was reported for the Japanese halfbeak *Hyporhamphus sajori* (Yu et al., 2016). Yu et al. (2016) suggested that the decline of surface seawater temperatures in the East Sea might have been caused by the blockage of the Tsushima Warm Current during the last glacial maximum, resulting in the local extinction of East Sea species and a subsequent substantial drop in genetic diversity, which is currently observed. *S*.* joyneri* would also have suffered similar demographic events, but probably more severely due to its preference for warmer habitats than *S*.* thompsoni* (Konishi & Nakabo, 2007). Therefore, Korean *S*.* thompsoni* comprised a single panmictic mitochondrial group, perhaps due to the survival of a single population in southern refugia, and a subsequent rapid recent expansion and migration to the East and Yellow Seas.

Unlike the results of mtDNA control region sequences (present study) and those of seven ms loci (Sekino et al., 2001), our results based on 11 msDNA loci revealed that there were two distinct clusters in Korean *S. thompsoni*. The occurrence ratio of the two clusters varied among sampling locations; for example, Cluster 2 was predominant in Korean coastal locations (the Eocheongdo, Chujado, and Yokjido Islands, and Sokcho), but Cluster 3 was predominant at Dokdo Island, and both clusters occurred in similar proportions at the Wangdolcho Reef. The Dokdo Island (offshore, in the middle of the East Sea) population was nearly identified as Cluster 3, differing from other local populations (average *F*_ST_ = 0.0468, *p* < .0009; Table 3); this result was supported by both STRUCTURE and DAPC results, although a few admixed individuals were found between clusters (Figures 2b, 3). An interesting result for the Wangdolcho Reef (between coast and offshore in the middle of the East Sea) population is that individuals belonging to two Korean *S*.* thompsoni* clusters were mixed in similar proportions (Cluster 2 in *S*.* thompsoni*: 0.466; and Cluster 3 in *S*.* thompsoni*: 0.524); this reef is geographically located in a transitional zone between the two clusters. These results provide the evidence of restricted gene flow between the Dokdo Island population and other local populations. The Wangdolcho Reef population may be a mixed population, not a hybrid population. AMOVA results also suggested that Korean *S*.* thompsoni* had diverged into two (ST_Eo, ST_Ch, ST_Yo, ST_So, and ST_Wa‐1 vs. ST_Wa‐2 and ST_Do), according to the F‐statistic values (*F*_CT_ = 0.1543, *p* < .009; *F*_SC_ = 0.0058, *p* > .009) (Table 4).

**Figure 5:**
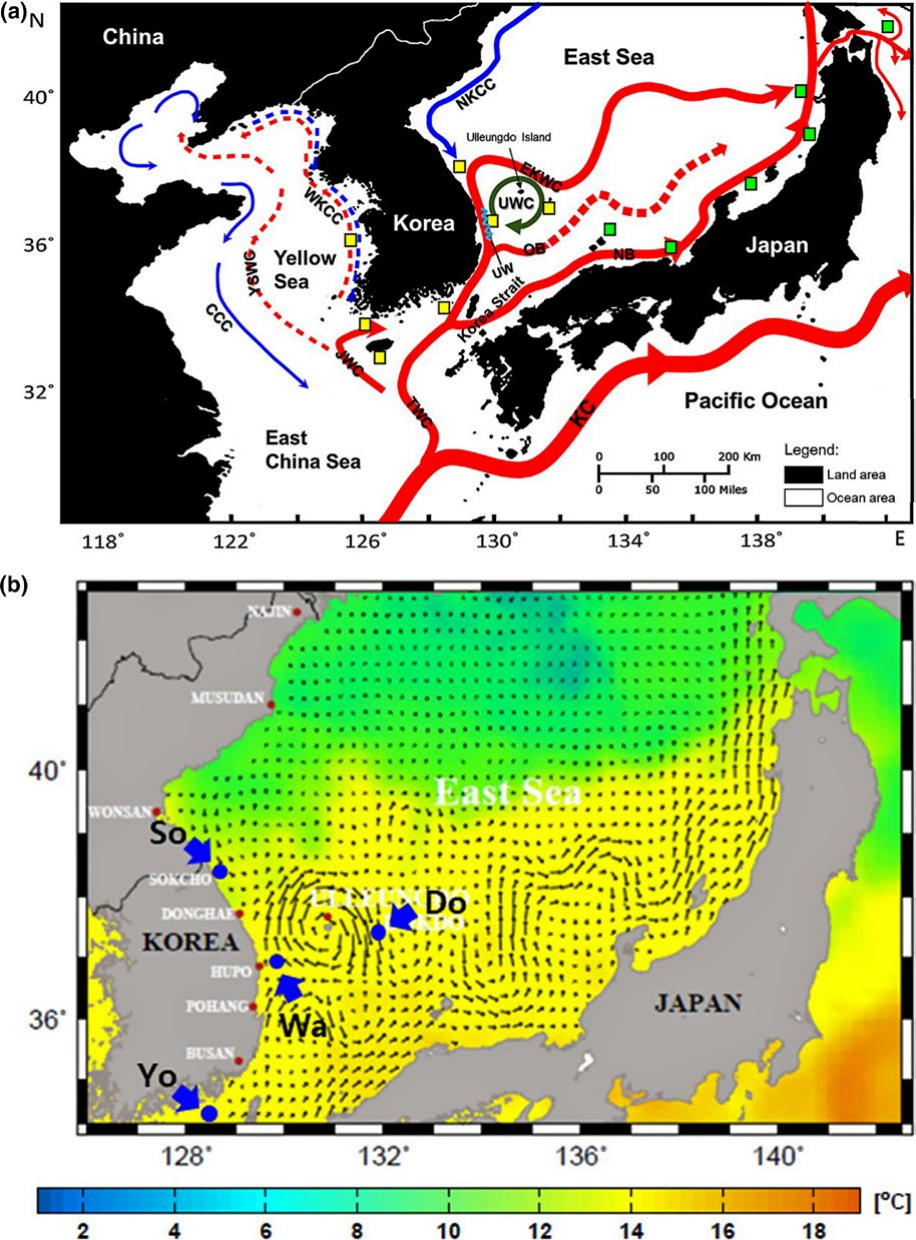
Major ocean currents around Korea and Japan (a). Yellow rectangles with black borders indicated the sampling location around Korea in this study, and green rectangles with black borders indicated the sampling location around Japan in the previous study (Sekino et al., 2001). Arrows and dashed lines represent ocean current flow, eddies, and upwelling. Red: warm current; blue: cold current; dark green: eddy; and sky blue: upwelling. CCC: Chinese Coastal Current; EKWC: East Korea Warm Current; JWC: Jejudo Warm Current; KC: Kuroshio Current; NB: Nearshore Branch of TWC; NKCC: North Korea Cold Current; OB: Offshore Branch of TWC; TWC: Tsushima Warm Current; WKCC: West Korea Coastal Current; YSWC: Yellow Sea Warm Current; UWE: Ulleung Warm Eddy; and UW: upwelling in western Wangdolcho Reef. Surface currents estimated from satellite altimeter data and satellite‐borne sea surface temperature (°C) in the East Sea at spring (March–May) 2015 (b). Large blue arrows and circles indicate sampling locations in the East Sea and Korea Strait. Yo: Yokjido Island; Wa: Wangdolcho Reef; Do: Dokdo Island; and So: Sokcho. Small black arrows indicate surface current directions and intensity (length). Data were obtained from the Korea Hydrographic and Oceanographic Agency (KHOA; http://www.khoa.go.kr)

Several studies have suggested that the genetic population structure of *Sebastes* species in the Northeast Pacific Ocean may result from discontinuity in gene flow driven by limitations on the dispersal of larvae and juvenile (often through the influence of various oceanographic factors). For example, the population structure of Pacific ocean perch (*Sebastes alutus*) based on ms loci showed the significant, geographically related genetic structure, which may be due to the limitation on the dispersal of larvae by oceanographic factors such as eddies (Palof, Heifetz, & Gharrett, 2011). Buonaccorsi et al. (2004) suggested that dispersal of larvae is also limited among grass rockfish (*Sebastes rastrelliger*) populations, and genetic differences among populations were significantly associated with geographic distance. In addition, patterns of population structure formation in the same geographic area have been observed in other rockfishes such as rosethorn rockfish (*Sebastes helvomaculatus*; Rocha‐Olivares & Vetter, 1999), blue rockfish (*Sebastes mystinus*; Burford, 2009), and vermilion rockfish (*Sebastes miniatus*; Hyde & Vetter, 2009). In case of *S. thompsoni* in the Northwest Pacific, long distance dispersal during the early life stages may also be highly influential in connecting very distant populations (Kokita & Omori, 1998, 1999; Nagasawa & Kobayashi, 1995). Sekino et al. (2001) demonstrated, in their molecular studies of Japanese *S*.* thompsoni* using seven msDNA loci, that low genetic divergence among far distant local populations was explained by the influence of larva and juvenile dispersal by the Tsushima Warm Current. Moreover, sampling was performed on the western coast of Japan, where the Tsushima Warm Current is so prevalent as to enable linkage between local populations. Conversely, this study detected significant population structure in Korean *S*.* thompsoni*, based on 11 ms loci, which may be associated with various ocean currents or eddies (e.g., the Tsushima Warm Current, East Korea Warm Current, North Korea Cold Current, Ulleung warm eddy, and Dokdo cold eddy). In spring, the strong Ulleung warm eddy (UWE), that is, the warm cyclonic eddy developed in the north or south of Ulleungdo Island, and the weak Dokdo cold eddy (DCE), that is, the cold anticyclonic eddy observed at the south of Dokdo Island, occur in the East Sea (Choi, Byun, & Lee, 2012; Lee & Niiler, 2005; Mitchell, Teague, Wimbush, Watts, & Sutyrin, 2005; Figure 5). These eddies flow between the Wangdolcho Reef and Dokdo Island, and may facilitate genetic exchange between them, while at the same time possibly precluding genetic exchange between Korean coastal populations and offshore populations (Figure 5). Furthermore, as UWE and DCE are in contact with each other and have opposite directions of rotation, dispersal flows more strongly toward Wangdolcho Reef. It is therefore presumed that larvae and juvenile of *S*.* thompsoni* released in Dokdo Island may be more actively transported toward Wangdolcho Reef. Some studies on the population genetics of *Sebastes* species have suggested that strong eddies restrict dispersal between local populations (Palof et al., 2011; Siegle, Taylor, Miller, Withler, & Yamanaka, 2013). According to Palof et al. (2011), it is likely that such eddies may contribute to genetic discontinuities by entraining or preventing pelagic larvae and juveniles from moving. Therefore, the strong eddies around the Ulleungdo and Dokdo Islands in the East Sea might cause self‐replenishment, suggesting a potential for allopatric differentiation over time.

Wangdolcho Reef, located 23 km offshore from the western margin of the East Sea, is influenced by two major currents (North Korean Cold current and East Korea Warm Current) with upwelling and small eddies, which occurs mainly along the western Reef (Lee & Myoung, 2003). Coastal upwelling acts as a physical barrier to larval dispersal for marine fishes (Graham, Field, & Potts, 1992), and also strongly divides water masses, enhancing the larval fish assemblage dichotomy (Tiedemann & Brehmer, 2017). Moreover, upwelling off the northeastern Pacific coastline determines the density of rockfish larvae and juveniles, and has a great effect on settlement and survival (Bjorkstedt, Rosenfeld, Grantham, Shkedy, & Roughgarden, 2002; Olivar, Sabatés, Pastor, & Pelegrí, 2016). Hence, it is assumed that oceanographic factors (e.g., upwellings, currents, and eddies) formed around the western Wangdolcho Reef influence the extent of dispersal during the early life cycle of *S*.* thompsoni* and are therefore potentially important in separating the eastern and western populations. The two clusters (Clusters 2 and 3) of individuals identified in Wangdolcho Reef may be roughly separated by physical barriers caused by upwelling to the west (mainly Cluster 2) and UWE (or DCE) to the east (mainly Cluster 3). Similar to our results, the community structure of phytoplankton around Wangdolcho Reef has shown a clear division into two areas, eastern and western (Shim et al., 2008), related to upwelling or eddies as mentioned above.

Although Sokcho is closer to Dokdo Island and Wangdolcho Reef than the other sampling locations in this study, the Sokcho population differed from these populations in the multiplex tests (e.g., STRUCTURE, DAPC, and AMOVA; Figures 2 and 3, Table 4). This is a good evidence of connectivity between the Korea Strait populations (i.e., the Yokjido and Chujado Islands) and the East Sea coastal population (Sokcho), which may be explained by a combination of (1) the physical characteristics of warm currents (such as Tsushima Warm Current and East Korea Warm Current) prevailing in the surface layer (Choi et al., 2012; Lee & Niiler, 2005) and (2) the biological characteristics of pelagic larvae and juveniles attaching to drifting seaweed (Kokita & Omori, 1998, 1999; Nagasawa & Kobayashi, 1995).

Most *S*.* thompsoni* populations in the Yellow Sea exhibit patchy distribution due to lack of suitable habitat and are exposed to the unique marine environments (semi‐enclosed, strong tidal current, shallow water, and high‐turbidity) and paleoclimatic change (Kim, 2009; Xu & Oda, 1999). These conditions may facilitate population subdivision due to limited gene flow and adaptive radiation (Gunderson, Vetter, Kritzer, & Sale, 2006; Mayer, Schiegg, & Pasinelli, 2009). However, this study did not detect any evidence of genetic variation between the Eocheongdo Island population (Yellow Sea) and other local populations. Some larvae released near the Jejudo and Chujado Islands might have been spread to the Yellow Sea with the help of the Tsushima or the Yellow Sea Warm Currents. Likewise, Japanese halfbeak (*Hyporhamphus sajori*; Yu et al., 2016) and Korean rockfish (*Sebastes schlegelii*; Gao et al., 2016; Zhang, Yanagimoto, Zhang, Song, & Gao, 2016), associated with drifting seaweed during early lifetime like *S. tompsoni*, in the similar geographic area showed no population structure based on mtDNA control region and ms loci, which may be attributable to the passive dispersal of the eggs and larvae through the Yellow Sea Warm Current. Extensive drifting seaweed, identified as *Sargassum horneri*, originated from the East China Sea during spring also flows into the Yellow Sea due to the effects of the Yellow Sea Warm Current and the northwestern monsoon (Hwang, Lee, Ha, & Park, 2016). Many larvae and juveniles associated with drifting seaweed might be transported to the Yellow Sea under these circumstances, thus allowing the Eocheongdo Island population to maintain high genetic homogeneity.

Our results indicate that the two subpopulations of Korean *S*.* thompsoni* may have differentiated primarily because of characteristics of the local marine environments and their consequent effects on the dispersal of larvae and juveniles, instead of through hybridization between *S*.* thompsoni* and *S*.* joyneri*. This study carefully suggests that Korean *S*.* thompsoni* needs to be managed at least two subpopulations, and further studies of the marine biogeographic boundary in the East Sea are needed for a more comprehensive understanding of metapopulation dynamics of marine species.

## CONFLICT OF INTEREST

None declared.

## AUTHOR CONTRIBUTIONS

H.J.Y and J.K.K conceived of and designed the study. H.J.Y performed laboratory work and analyzed the data. H.J.Y and J.K.K wrote the manuscript.

## DATA ACCESSIBILITY

DNA sequences are accessible at GenBank accession numbers: MF817322‐MF817441. Sampling locations and microsatellite genotypes are available at Dryad https://doi.org/10.5061/dryad.2024j.
