# Supplementary material for: Upwelling and eddies affect connectivity among local populations of the goldeye rockfish, Sebastes thompsoni (Pisces, Scorpaenoidei)
Source: Ecol Evol. 2018 Apr 2;8(9):4387–402. doi: 10.1002/ece3.3993 (PMC5938445; doi:10.1002/ece3.3993)
Supplement: Supplementary file 1 [file ECE3-8-4387-s001.docx]

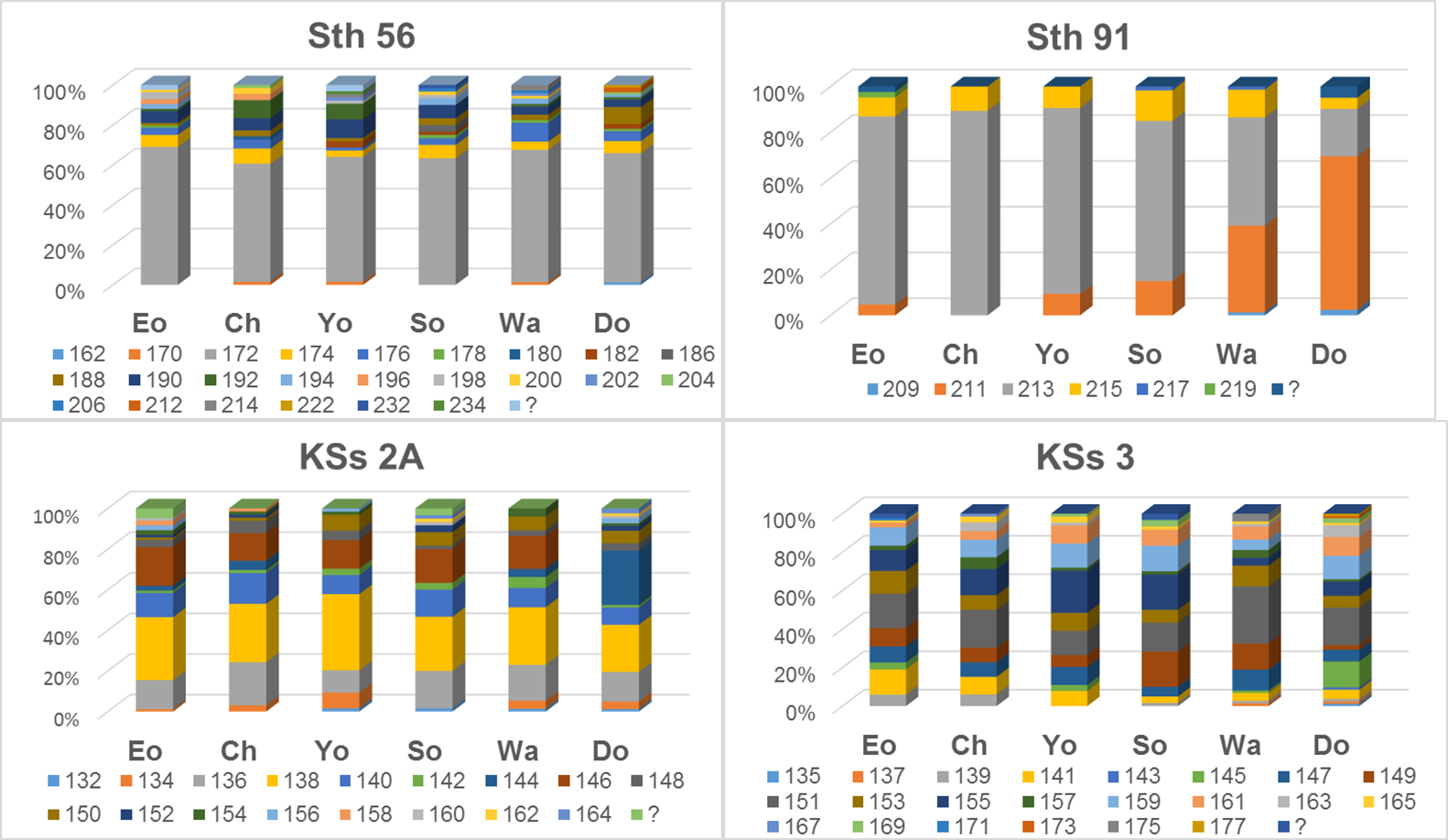


**Fig. S1.** Allele frequency distributions for *Sebastes thompsoni* populations at six sampling locations, based on four microsatellite loci showing differences between populations.


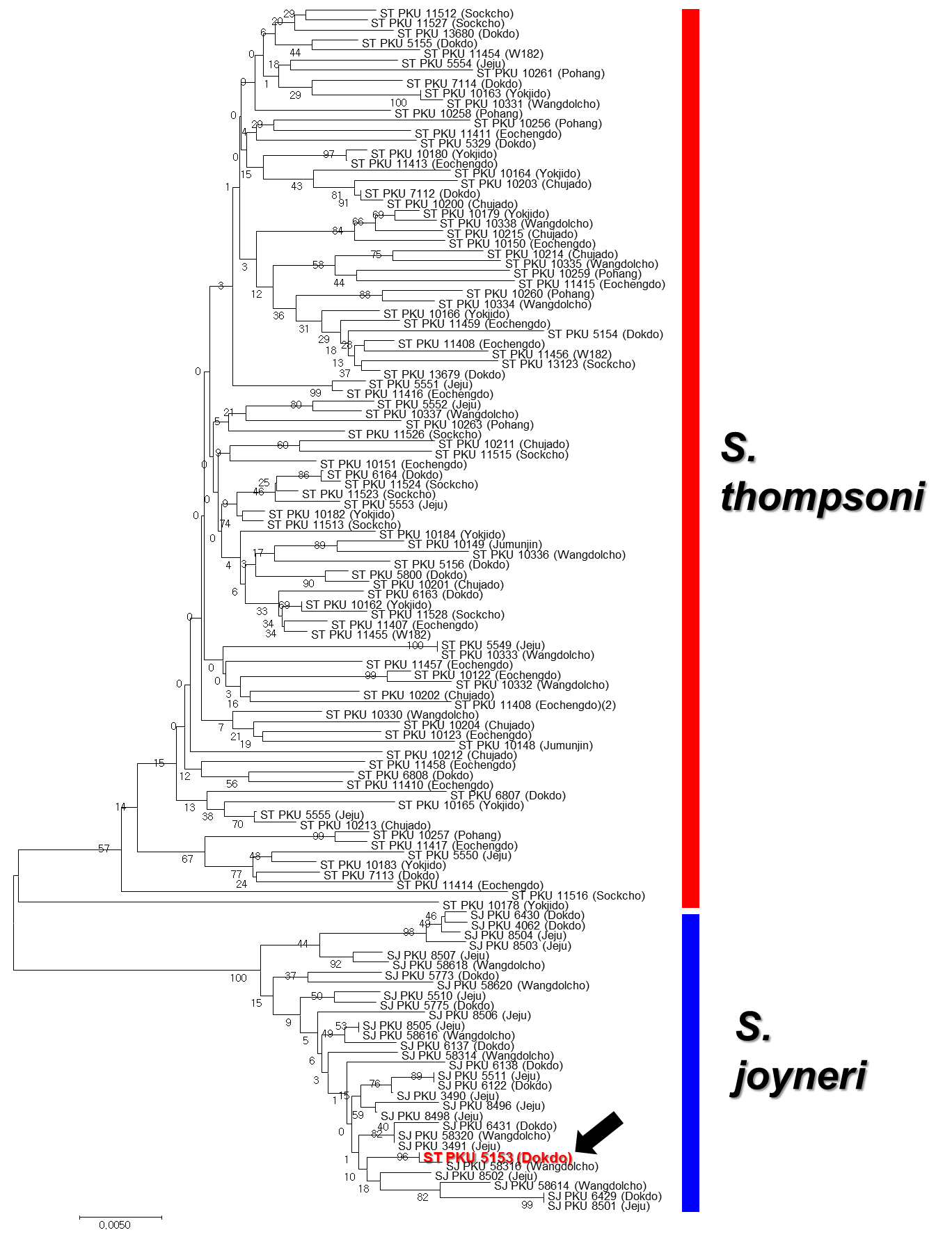


**Fig. S2.** Neighbor-joining (NJ) tree based on mtDNA control region sequences, showing the relationships between *S*. *thompsoni* (ST) and *S*. *joyneri* (SJ). Numbers at branches indicate bootstrap probabilities for 1,000 bootstrap replications. Bar indicates a Tamura–Nei genetic distance (Tamura & Nei, 1993) of 0.005. Black arrow indicates an individual identified as *S*. *thompsoni* by its morphological characteristics.


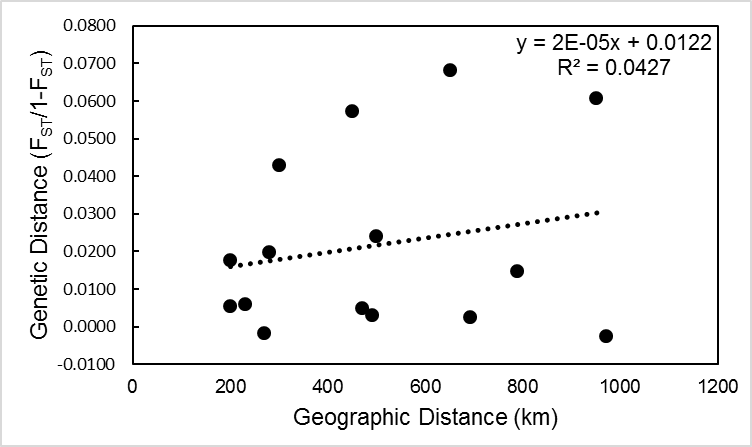


**Fig. S3.** The relationship between genetic distance (F_ST_/1–F_ST_) and geographic distance within *S*. *thompsoni* populations for all pairwise sampling locations derived from allele frequencies of 11 microsatellite loci.
